# The problem with peripherally inserted central catheters in China

**DOI:** 10.12669/pjms.293.3741

**Published:** 2013

**Authors:** Zhoupeng Wu, Jichun Zhao

**Affiliations:** 1 Zhoupeng Wu, Department of Vascular Surgery, West China Hospital, Sichuan University, China.; 2 Jichun Zhao, Department of Vascular Surgery, West China Hospital, Sichuan University, China.

Peripherally Inserted Central Catheters (PICCs) provide more venous access for tasks for delivery of medicine, laboratory testing and hemodynamic monitoring which occupy a fundamental role in the treatment of seriously ill patients, specially in the diagnosed tumor patients. However, despite their many benefits, PICCs are not innocuous and associated with important complications. We must pay attention to the upper extremity deep venous thromboembolism.

Although the use of central venous catheters (CVCs) are widespread in intensive care unit (ICU) and non-ICU in most hospitals, PICCs have become more popular in non-ICU department.^1^ There are many advantages in this technique compared with CVCs. First of all, these devices are safer than CVCs which could eliminate the discomfort associated with phlebotomy, they also provide extended and reliable venous access. Then, they reduce the rates of central line-associated bloodstream infection (CLABSI) significantly. Furthermore nurses can get special training to acquire the technique which can place PICCs at the patients bedside. These factors will lead to reduced hospitalization cost to the patients enabling them to get intravenous therapy at home. All over the world, the patients have welcomed and supported the widespread use of these venous catheters. Increased use of PICCs in China and triggered the debate regarding PICCs’ security and effectiveness.

There are some literatures about PICC and UEDVT reported in many countries apart from China. But there are few article in China. Xing L et al^2^ reported that there were 187 breast cancer patients using a PICC for chemotherapy from August 2009 to July 2011. Four of them were removed as a result of a PICC-related UEDVT.^2^ In the past one year, in our hospital, medical records of 126 consecutive patients who underwent upper extremity venous duplex ultrasound (VDU) had acute UEDVT. About 74% patients had arm swelling or arm pain; 93% had cancer; 96% had inserted PICC. Cancer patients with PICC had more occurred UEDVT. At the same time, 13% patients also had lower extremity deep vein thrombosis. The incidence of pulmonary embolism screened by the computed tomography angiography (CTA) was 7% with 9 patients and one month mortality rate was 5.5%. The majority of patients (90%) with UEDVT received anticoagulation therapy and 10% had no anticoagulation because of anticoagulation taboo. The most common risk factors for UEDVT were PICC and a diagnosis of cancer. The rate of PE and mortality rate from UEDVT made no difference at 7% and at 5.5%.

There are no clear guidelines for patients at risk of UEDVT associated with PICC. Future research in Chaina should focus on risk evaluation and management for patients at risk of UEDV.
